# Identification and validation of USP15 and CUL2 as ubiquitination related biomarker in chronic obstructive pulmonary disease

**DOI:** 10.1186/s41065-025-00460-1

**Published:** 2025-05-24

**Authors:** Shulei Sun, Zhaoxiong Zhang, Haiyan Zhao

**Affiliations:** 1 Department of Respiratory and Critical Care Medicine, Tianjin Medical University General Hospital, 154 Anshan Road, Heping District, Tianjin, 300052 China; 2 Tianjin Medical University General Hospital, Tianjin, 300052 China

**Keywords:** Ubiquitination related genes, COPD, Bioinformatics analysis, USP15, CUL2

## Abstract

**Purpose:**

Ubiquitination is one of the important epigenetic modifications, influencing the development of various diseases. The objective of this study is to investigate the ubiquitination related genes in chronic obstructive pulmonary disease (COPD).

**Methods:**

The gene microarray dataset from COPD patients and ubiquitination related genes were analyzed. Venn diagram analysis was used to intersect differentially expressed genes and ubiquitination related genes. The functional enrichment analysis of Gene Ontology (GO), Kyoto Encyclopedia of Genes and Genomes (KEGG) and Gene Set Enrichment Analysis (GSEA) were performed on differentially expressed ubiquitination related genes. Finally, we confirmed the expression of hub genes through qPCR and western blot experiments in clinical COPD patients and cell lines.

**Results:**

We identified 2,932 differentially expressed genes and 96 differentially expressed ubiquitination related genes. GO analysis indicated that the differentially expressed ubiquitination related genes were mainly enriched in post-translational protein modification and ubiquitin ligase complex. KEGG analysis showed that ubiquitination related genes were mainly involved in ubiquitin mediated proteolysis and TNF signaling pathway. GSEA analysis suggested that some hub genes are involved in allograft rejection, IL6/JAK/STAT3 signaling and inflammatory response. Our qPCR and western blot experimental results indicate that the expression of USP15 and CUL2 is higher in COPD group compared to the control group, consistent with the bioinformatics analysis.

**Conclusion:**

Our bioinformatics analysis and experimental results suggest that USP15 and CUL2 may contribute to the progression of COPD through ubiquitination modification. To our knowledge, this is the first study to demonstrate the involvement of USP15 and CUL2 in COPD. Our results may provide new insights into the diagnosis and treatment of COPD.

**Supplementary Information:**

The online version contains supplementary material available at 10.1186/s41065-025-00460-1.

## Background

Chronic obstructive pulmonary disease (COPD) is a global condition distinguished by irreversible airflow obstruction and ongoing respiratory symptoms [1]. The foundation of COPD etiology lies in airway remodeling, which is closely associated with the inflammation, repair, or structural changes within the airway epithelium [2, 3]. Chronic obstructive pulmonary disease (COPD) stands as a major global health challenge, significantly impacting morbidity and the demand for healthcare services [4]. However, most current COPD treatments focus on relieving symptoms and preventing acute exacerbations. Therefore, it is necessary for us to explore the pathological mechanisms of COPD, which may provide assistance for the diagnosis and treatment of COPD.

Ubiquitination is a protein modification process that involves attaching ubiquitin molecules to target proteins, marking these proteins for degradation or regulating their activity [5, 6]. Ubiquitination is essential for maintaining the balance and function of proteins within cells and is involved in regulating various cellular activities, including cell proliferation [7, 8]. Dysfunction of the ubiquitin system is related to the pathophysiology of multiple diseases. For example, one study reported that TRIM40 inhibits IgA1-induced proliferation of glomerular mesangial cells by inactivating the NLRP3 inflammasome through ubiquitination [9]. Another study showed that PRMT6 facilitates EZH2 protein stability by inhibiting TRAF6-mediated ubiquitination degradation, thereby promoting the invasion and migration of glioblastoma cells [10]. However, ubiquitination related genes have not been reported in COPD.

The process of ubiquitination involves multiple steps, and many genes are related to ubiquitination. The main objective of this study is to explore the ubiquitination related genes in COPD. Firstly, we obtained differentially expressed ubiquitination related genes in COPD by analyzing the GSE38974 database [11]. Furthermore, the potential functions of ubiquitinated differentially expressed genes were identified through DEG analysis using GO, KEGG, and GSEA. Finally, the expression of USP15 and CUL2 in COPD patients and cell lines was performed by qPCR and western blot. By analyzing ubiquitination related genes in COPD, we hope to provide assistance in the development of diagnostic markers and potential therapeutic targets for COPD.

## Materials and methods

### GEO dataset

The mRNA expression profile dataset GSE38974 was obtained from the Gene Expression Omnibus (GEO, http://www.ncbi.nlm.nih.gov/geo/) database utilizing the R package GEOquery (version 2.74.0). The GSE38974 dataset is based on the GPL4133 platform, comprising a total of 32 samples, which consists of 23 COPD samples and 9 control samples.

### Differentially expressed genes (DEG) and ubiquitination related genes

For detecting the differentially expressed genes between the COPD patients and control groups, an analysis was conducted using the limma package (version 3.62.1), applying a criterion of adjusted *P*-value below 0.05 alongside an absolute log2 fold change greater than 0.5. For the visualization of the results, the ggplot2 package (version 3.5.1) was employed to construct a volcano plot. Ubiquitination related genes were downloaded from the MSigDB database (https://www.gsea-msigdb.org/gsea/index.jsp). After searching the database, we obtained a total of 742 ubiquitination related genes. The differential expressed ubiquitination related genes were obtained through Venn diagram analysis. Intersection genes were included in subsequent analyses.

### Gene set enrichment analysis (GSEA) and Single-gene GSEA

GSEA helps in discovering sets of genes potentially linked to a disease, which are either upregulated or downregulated in a given data set. GSEA analysis was also conducted using the “clusterProfiler” package (version 4.14.4) in R, primarily utilizing the " h.all.v2024.1.Hs.symbols.gmt” from the Molecular Signatures Database (MSigDB) database. Single-gene GSEA was conducted to explore the potential roles of hub genes utilizing the “clusterProfiler” package. Samples were categorized into high-expression and low-expression groups according to the median expression levels of the hub genes.

### Protein-protein interaction (PPI) analysis

The PPI analysis of ubiquitination related genes was conducted using the STRING database with a composite score threshold set at ≥ 0.4. The resulting data was imported into cytoscape software (version 3.6.1) for network graph visualization.

### GO and KEGG analysis

The differential expressed ubiquitination related genes were analyzed through Gene Ontology (GO) analysis and Kyoto Encyclopedia of Genes and Genomes (KEGG) pathway analysis to uncover the biological functions and signaling pathways associated with the initiation and progression of the disease. The analysis of Gene Ontology mainly involves the enrichment of biological processes, cellular components, and molecular functions.

### Correction analysis and transcription factor (TF) -hub gene network

The expression correlation of some hub genes was analyzed using the “corrplot” package (version 0.95) of R software. In addition, the potential transcription factors of hub genes were predicted through NetworkAnalyst database (https://www.networkanalyst.ca/). Subsequently, the prediction results were visualized using cytoscape software.

### qPCR

We collected peripheral blood samples from 6 COPD patients and 6 healthy controls at the Tianjin Medical University General Hospital. This study was approved by and conformed to the by the Medical Ethics Committee of Tianjin Medical University General Hospital. COPD was defined according to the Global Initiative for Chronic Obstructive Lung Disease (GOLD) criteria, as post-bronchodilator FEV_1_/FVC < 70% and presence of chronic respiratory symptoms such as cough or dyspnea. Patients with other respiratory diseases, including asthma, tuberculosis, lung cancer were excluded. Peripheral blood total RNA was extracted using an RNA extraction kit. The RNA was subsequently reverse transcribed into cDNA using a cDNA Reverse Transcription Kit. Real-time quantitative PCR was performed using the SYBR Green PCR Master Mix. The primers used for qPCR are shown in Table 1. The data were analyzed using the 2^−ΔΔCT^ method, employing β-actin as the internal control.

**Table 1:** Primer sequences used for qPCR

Primer	5’-3’
*USP15*-qPCR-F *USP15*-qPCR-R *CUL2*-qPCR-F *CUL2*-qPCR-R *ACTB*-qPCR-F *ACTB*-qPCR-R	TCAAAATGTGTATCCTGGACCCA GTGCTATTGGCTCTTGACCTT ACGACAATAAAAGCCGTGGTC GGATAGGCCACACATAAAGCAT TGGCACCCAGCACAATGAA CTAAGTCATAGTCCGCCTAGAAGCA

### 16HBE cells culture and treatment

The human lung bronchial epithelial cell line (16HBE) was cultured in PRMI 1640 medium containing 10% fetal bovine serum at 37 °C in a 5% CO_2_ atmosphere. The cigarette smoke extract (CSE) was freshly prepared using a vacuum extractor to extract cigarette smoke. The CSE was diluted to a concentration of 5% with PRMI 1640 medium. 16HBE cells were treated with 5% CSE for 48 h and then used for subsequent experiments.

### Western blot

Total protein was extracted using RIPA lysis buffer. The protein concentration was determined with the BCA protein assay kit. The protein lysates were separated by SDS-PAGE and subsequently transferred to PVDF membranes. The PVDF membranes were incubated with specific antibodies overnight at 4 °C, followed by incubation with the secondary antibody at room temperature for 2 h. Finally, the proteins were visualized using a chemiluminescence kit. The specific antibodies mainly consist of USP15 (Proteintech, China), CUL2 (Proteintech, China) and β-actin (Proteintech, China).

### Statistical analysis

Statistical analyses were conducted using R (version 4.4.1) software. A two-tailed *P*-value < 0.05 was considered statistically significant.

## Results

### Identification the differentially expressed genes of COPD

**Fig. 1:**
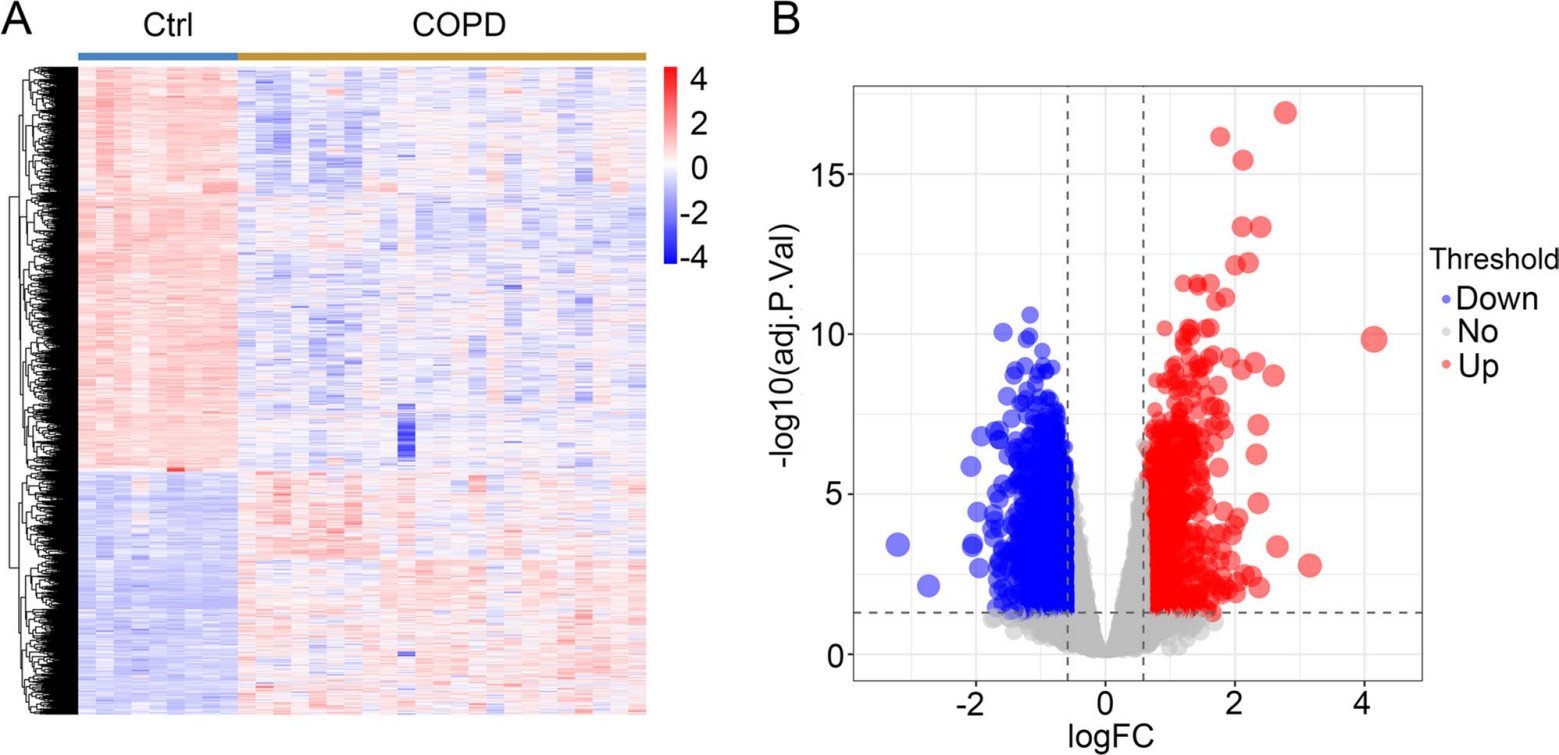
Differentially expressed genes between the COPD patients and the healthy samples. (**A**) Heatmap visualization of differentially expressed genes in COPD compared to normal groups. (**B**) Volcano plot illustrating the differentially expressed genes between the COPD and normal groups

To explore the differentially expressed genes in COPD, we analyzed the GSE38974 database. As shown in Fig. 1A and B and 2932 differentially expressed genes were screened and visualized using volcano and heat maps, including 1099 upregulated genes and 1833 downregulated genes.

### GSEA analysis of COPD

**Fig. 2:**
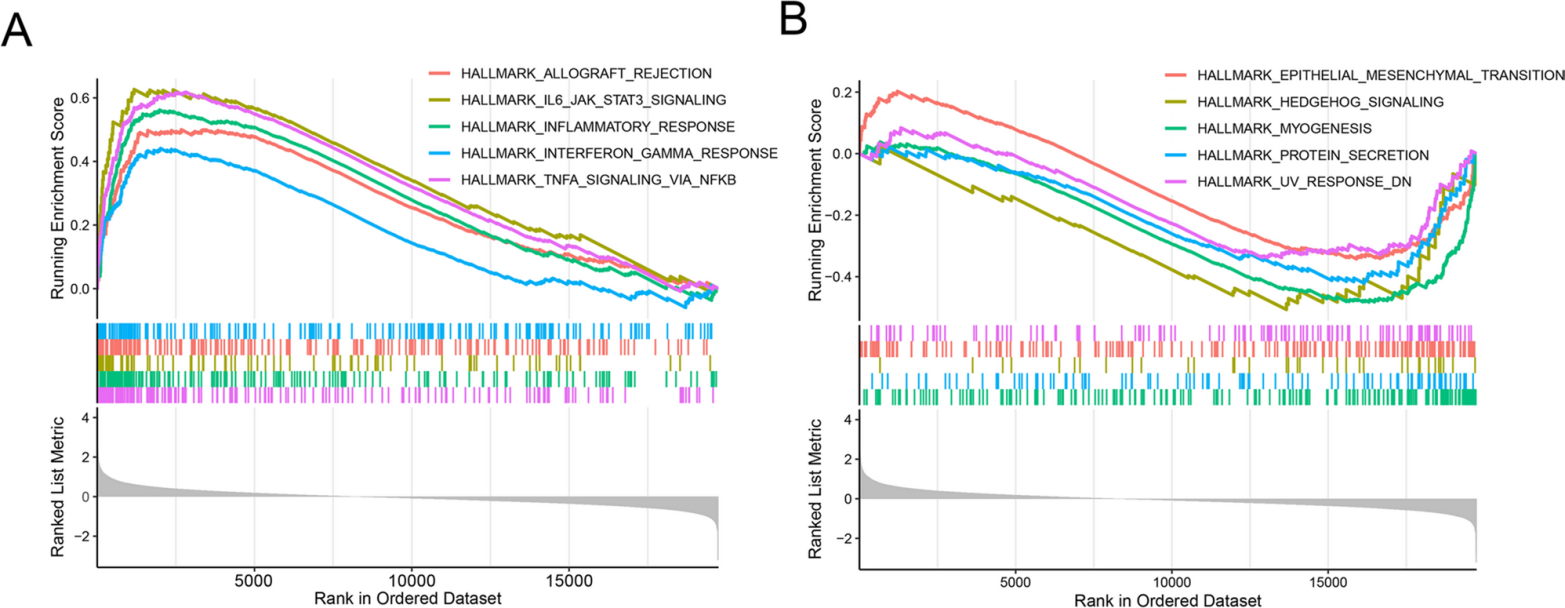
GSEA analysis between COPD patients and healthy control groups. (**A**) Top five positively enriched terms in the COPD group compared to the normal group. (**B**) Top five negatively enriched terms in the COPD group compared to the normal group

To further investigate the potential functional differences between COPD and the normal control group, we conducted GSEA analysis. The results indicated that the top five positively enriched terms mainly include the allograft rejection, IL6/JAK/STAT3 signaling and inflammatory response. In addition, the top five negatively enriched terms mainly include epithelial mesenchymal transition, hedgehog signaling, myogenesis and protein secretion (Fig. 2A and B).

### Expression of ubiquitination related genes in COPD

**Fig. 3:**
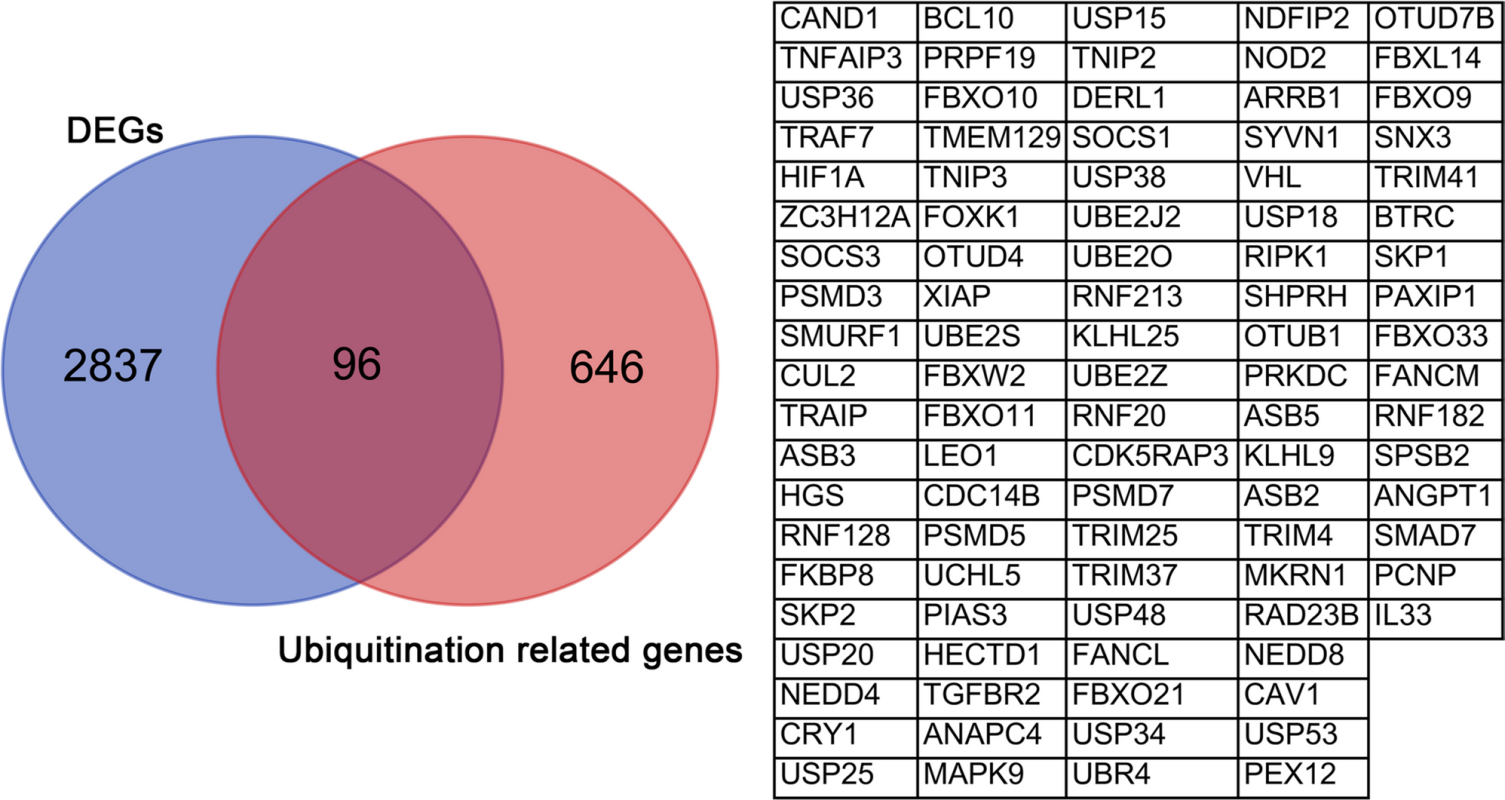
Venn diagram illustrating the overlap between differentially expressed genes and ubiquitination related genes

**Fig. 4:**
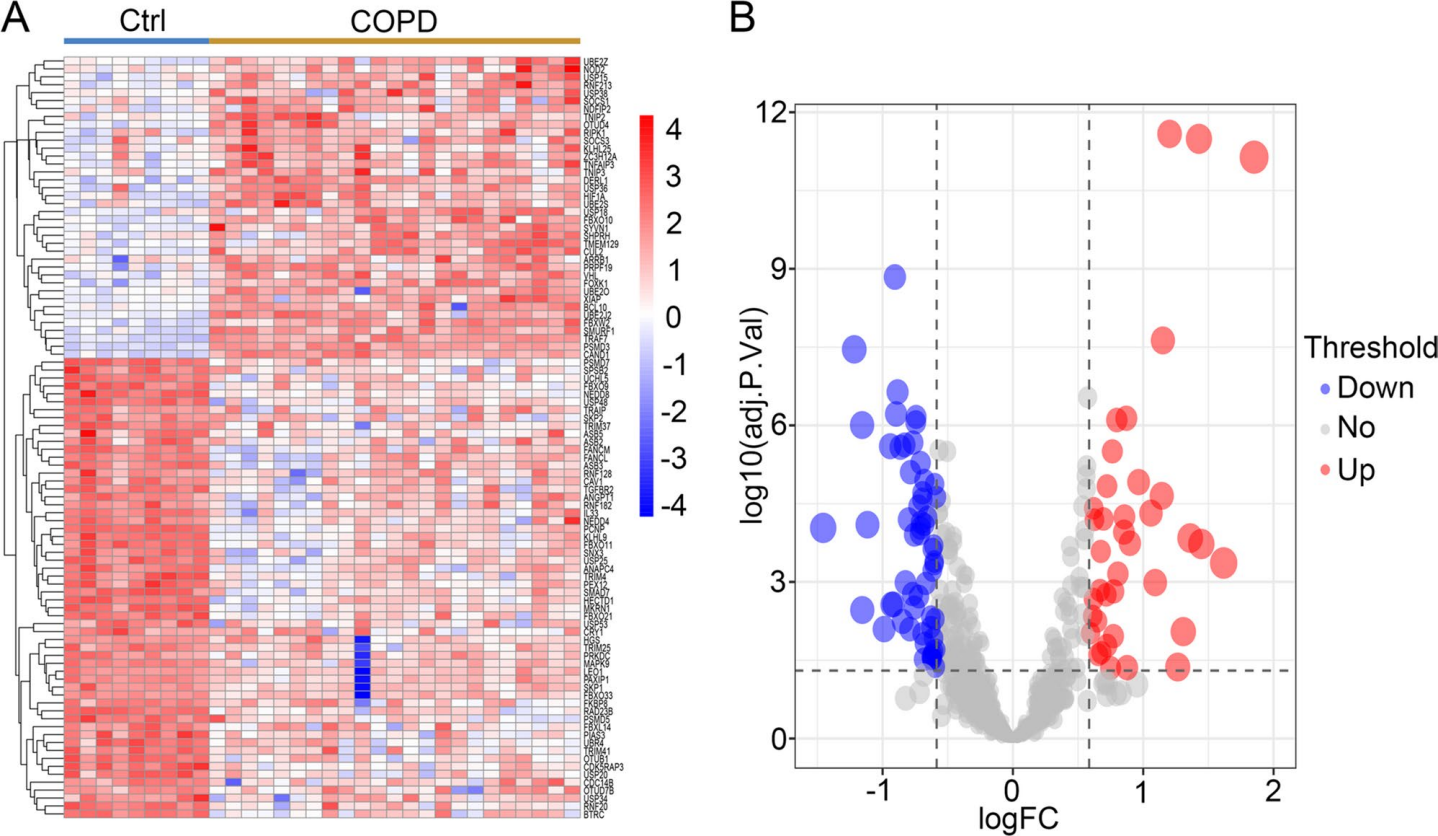
Differentially expressed ubiquitination related genes between the COPD samples and normal samples. (**A**) Heatmap analysis. (**B**) Volcano plot analysis

To further obtain ubiquitination related genes associated with COPD, we downloaded 742 ubiquitination related genes from Molecular Signatures Database. We used a Venn diagram to find the intersection of differentially expressed genes and ubiquitination related genes. The results indicated that there are 96 differentially expressed ubiquitination related genes in COPD (Fig. 3). To further analyze the expression of these 96 genes in COPD, we used heatmaps and volcano plots for visualization. The results indicated that there were 38 upregulated genes and 58 downregulated genes (Fig. 4A and B).

### GO and KEGG enrichment analysis of ubiquitination related genes

**Fig. 5:**
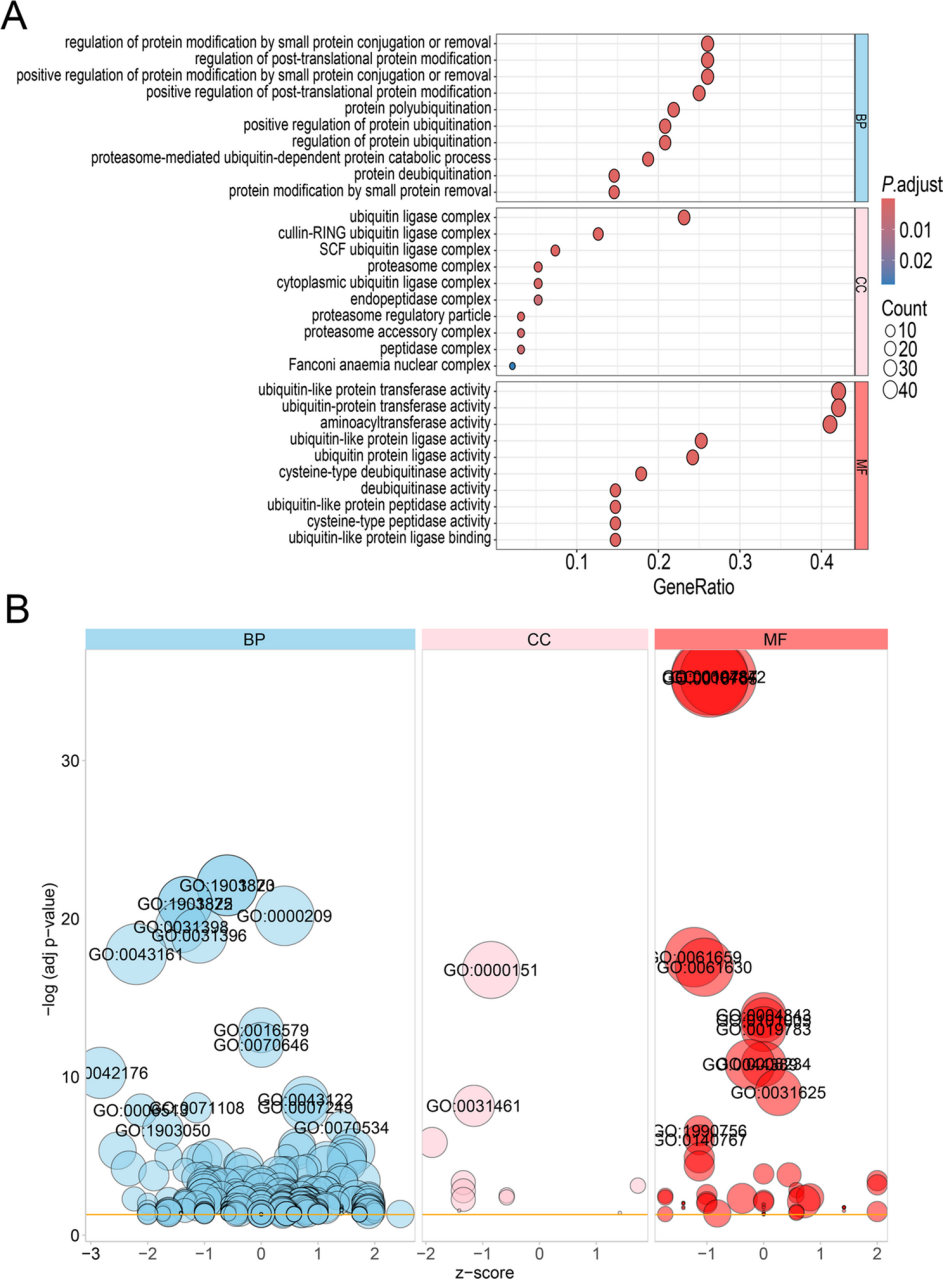
Gene Ontology (GO) analysis of ubiquitination related genes in COPD. (**A)** and (**B)** Bubble plot of enriched GO terms

**Fig. 6:**
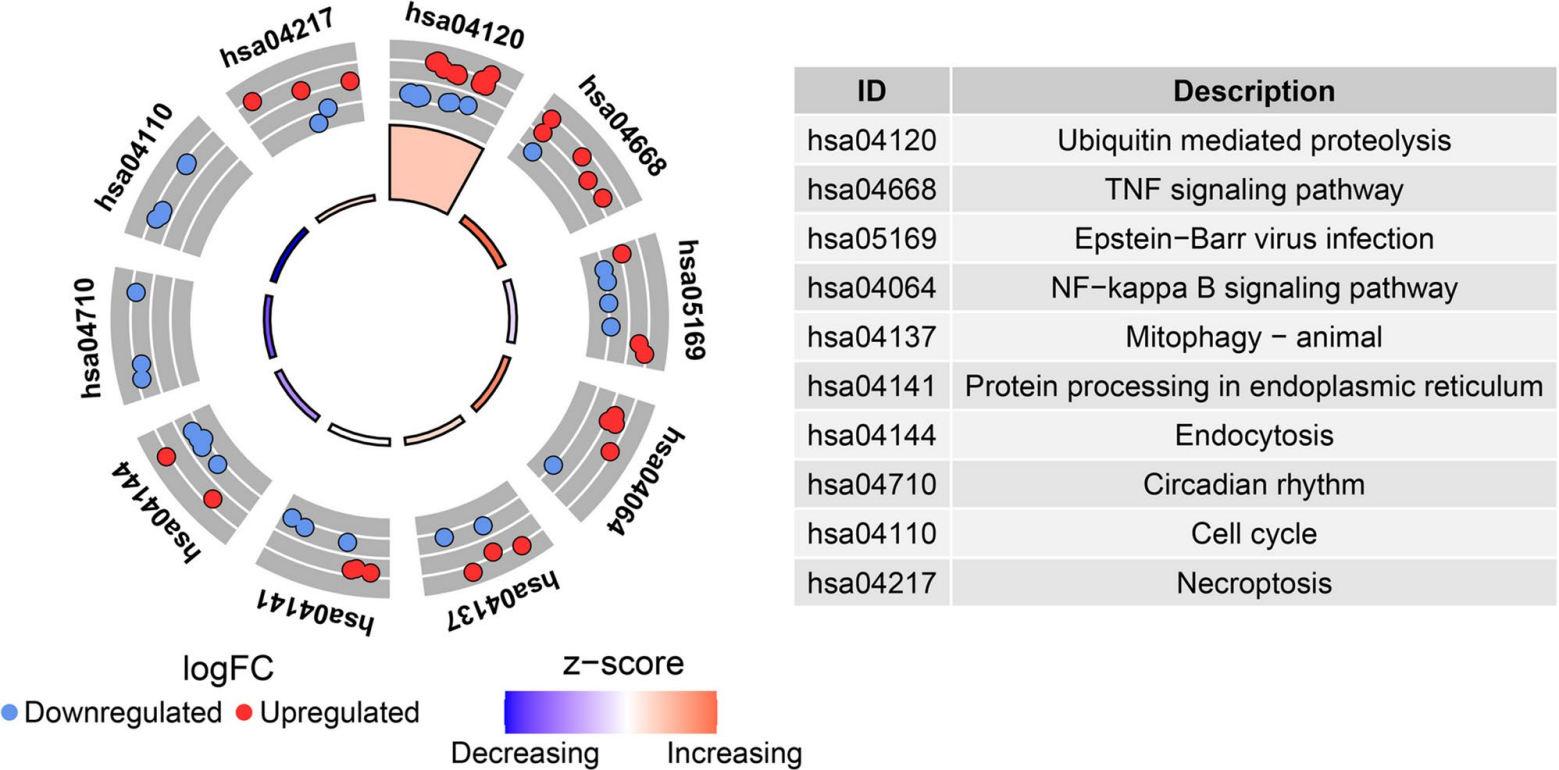
Kyoto Encyclopedia of Genes and Genomes (KEGG) analysis of ubiquitination related genes in COPD

To further analyze the potential functions of the 96 ubiquitination related genes in COPD, we performed GO and KEGG analyses. The GO results displayed the biological process (BP), cellular component (CC) and molecular function (MF) terms of the ubiquitination related genes. The biological process terms enriched in regulation of protein modification by small protein conjugation or removal, regulation of post-translational protein modification and protein polyubiquitination. The cellular component terms enriched in ubiquitin ligase complex, cullin-RING ubiquitin ligase complex and SCF ubiquitin ligase complex. The molecular functions terms enriched in ubiquitin-like protein transferase activity, ubiquitin-protein transferase activity and ubiquitin protein ligase activity (Fig. 5A and B; Supplementary Table S1). KEGG analysis showed that ubiquitination related genes were mainly involved in ubiquitin mediated proteolysis, TNF signaling pathway and protein processing in endoplasmic reticulum (Fig. 6; Supplementary Table S2).

### Protein-protein interaction of COPD

**Fig. 7:**
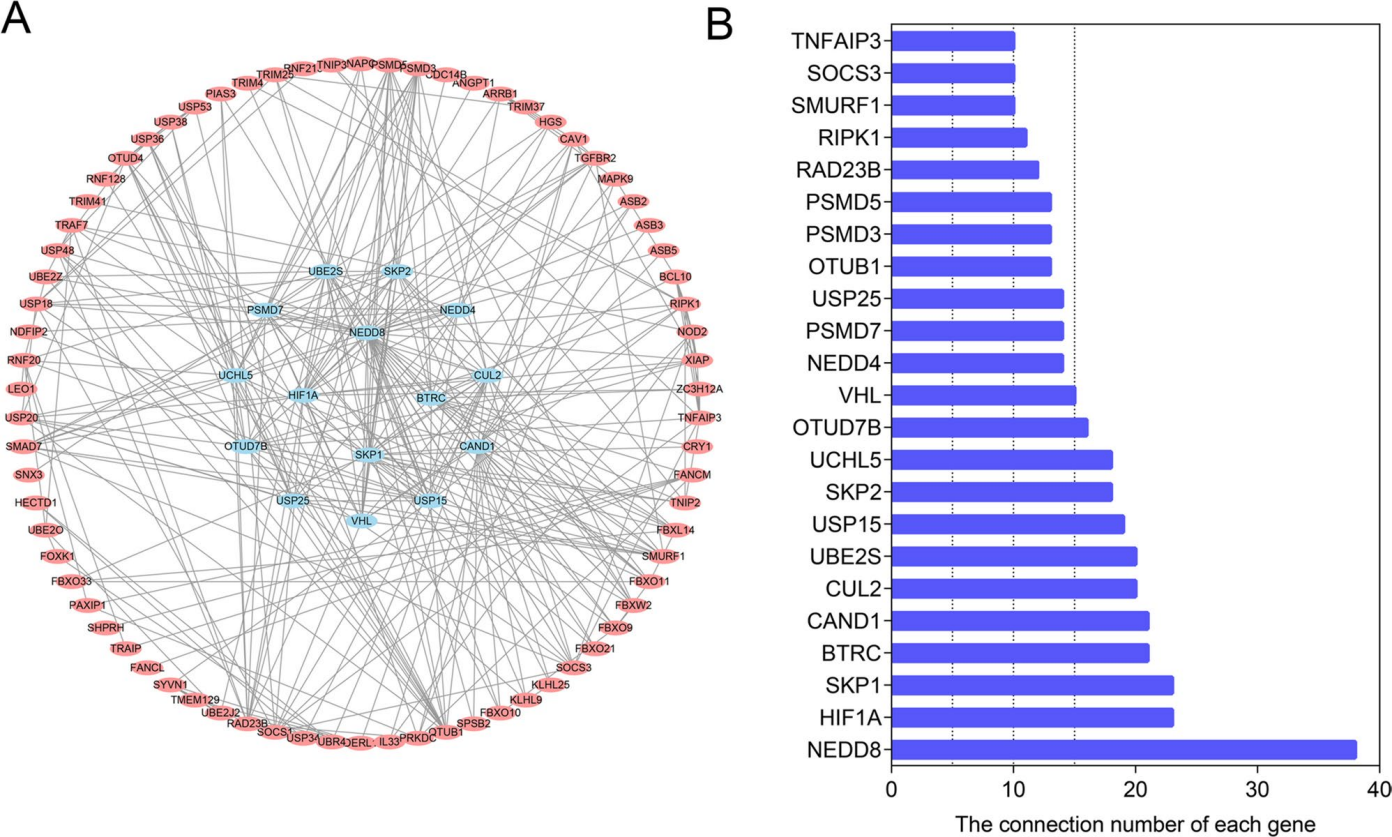
Protein-protein interaction (PPI) among proteins encoded by ubiquitination related genes. (**A**) Protein-protein interaction network. (**B**) The interaction count of the genes

**Fig. 8:**
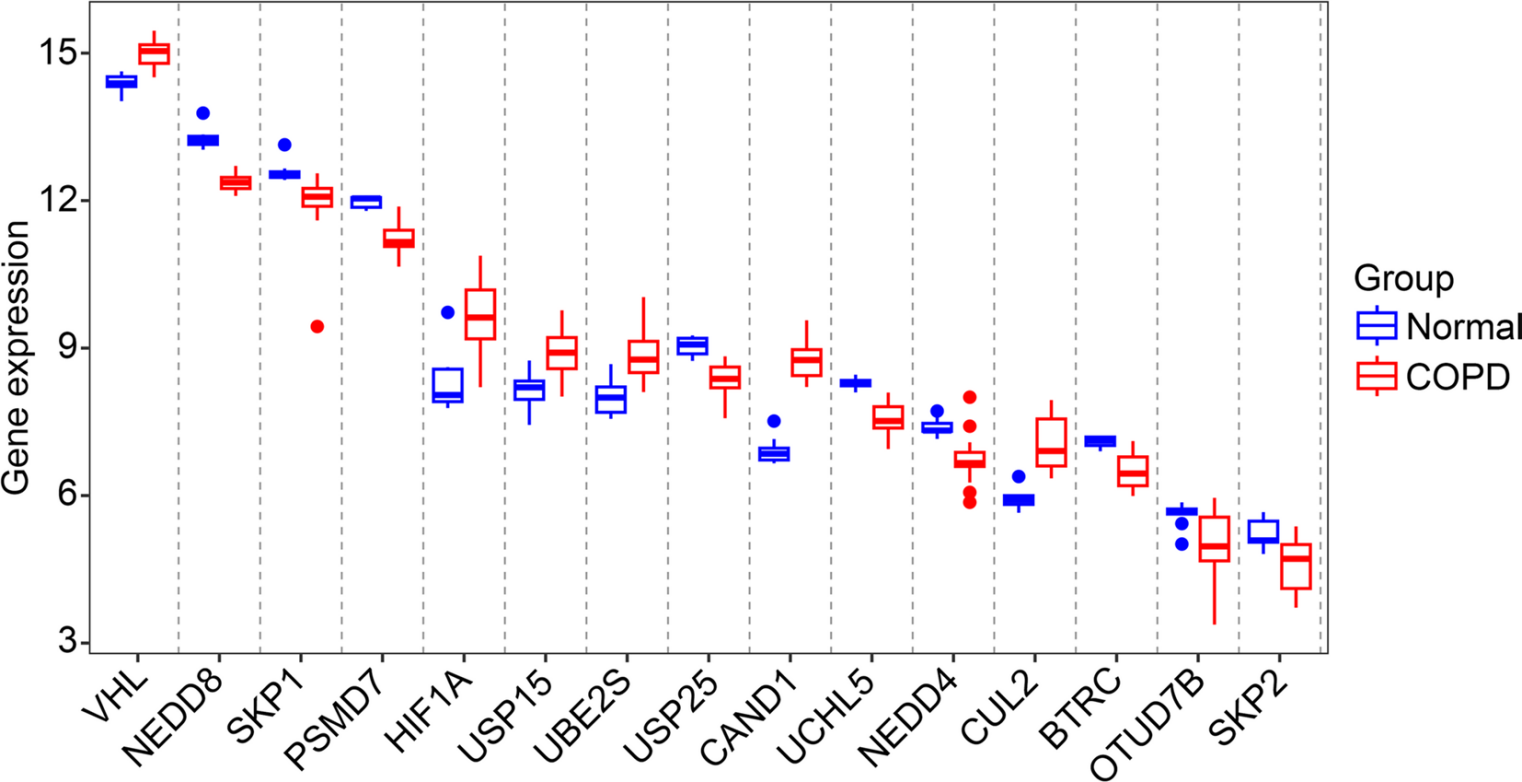
The box plot of 15 hub ubiquitination related genes in COPD and healthy samples

To evaluate the potential interactions of these ubiquitination related genes, we performed PPI analysis. The results revealed the interaction network and interaction count of the proteins encoded by these ubiquitination related genes (Fig. 7A and B). Based on the number of interactions, we defined the top 15 as hub genes. The box plot demonstrated the expression of these genes in COPD and healthy controls (Fig. 8).

### Correlation analysis and transcription factor of hub genes

**Fig. 9:**
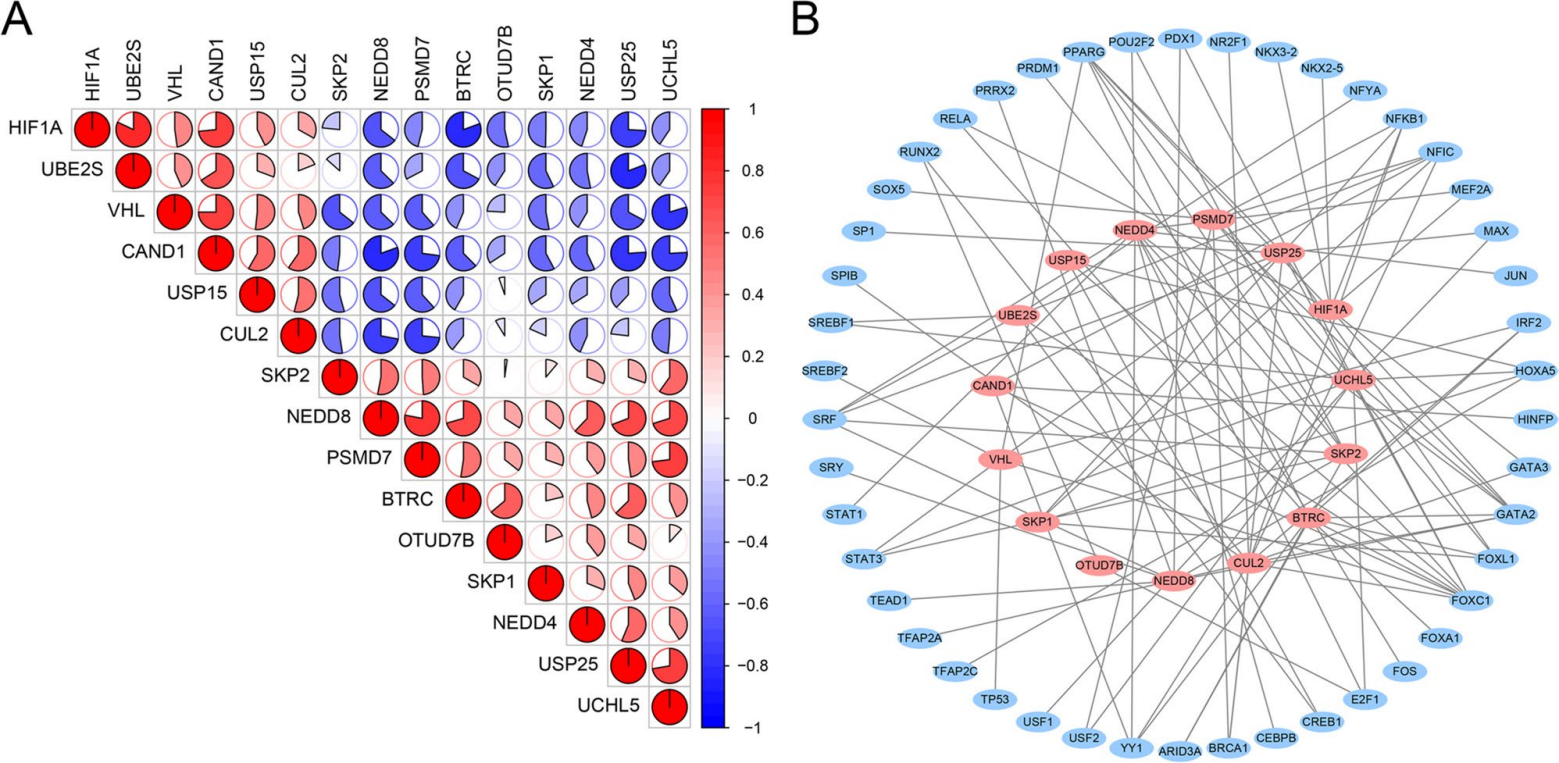
Correlation analysis and potential transcription factors of hub genes. (**A**) Correlation analysis of the 15 ubiquitination related genes. (**B**) Interaction network between transcription factors and hub genes

To further explore the expression correlation of these hub genes, we performed a correlation analysis. The results in Fig. 9A indicated that red circles suggest a positive correlation between the expression of two genes, while blue circles indicate a negative correlation. In addition, we predicted the potential upstream transcription factors for these 15 hub genes, and the results indicated that the transcription factors included FOXC1, GATA2, PPARG, SRF and HOXA5 (Fig. 9B).

### Single-gene GSEA of hub genes

**Fig. 10:**
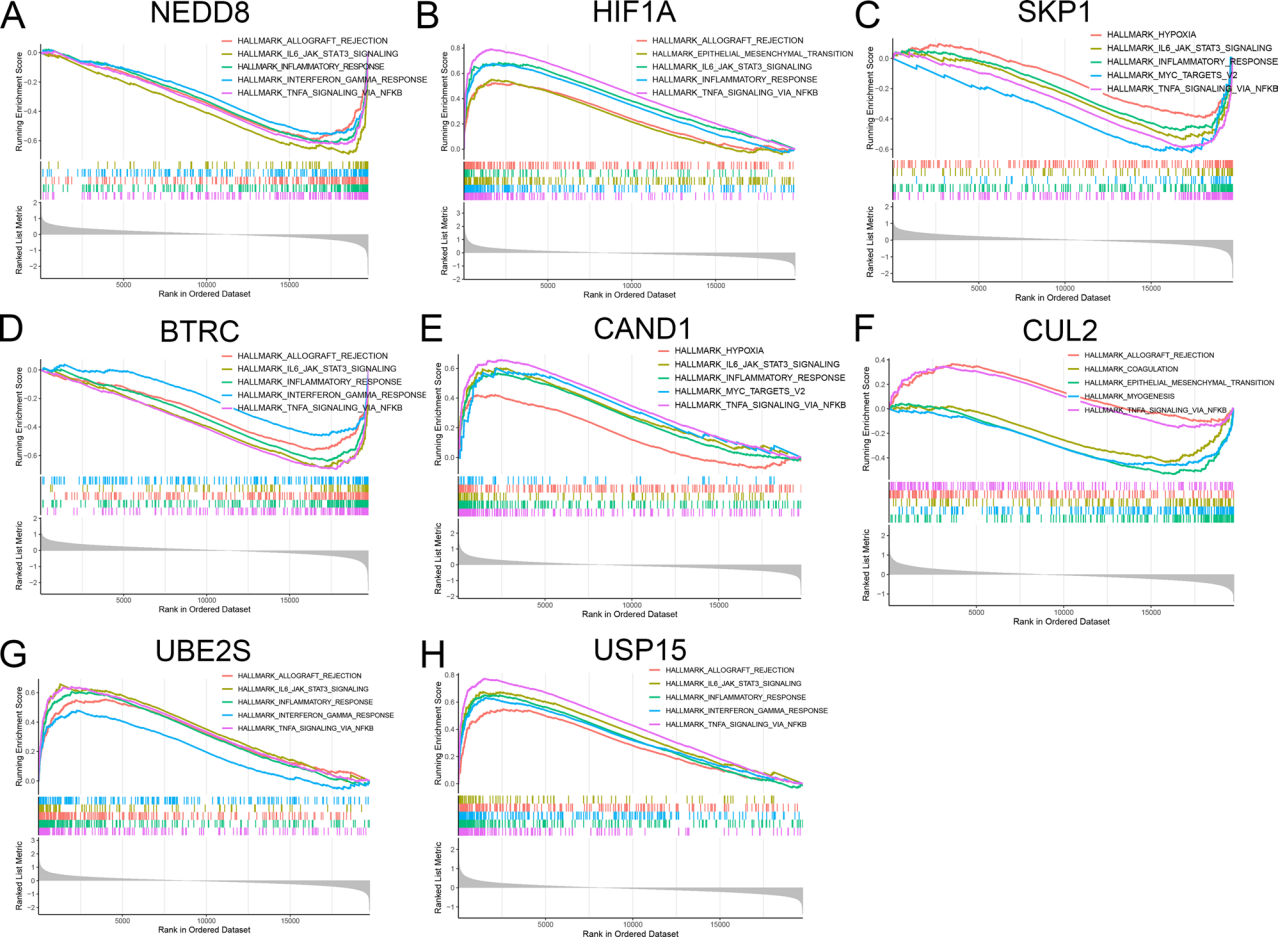
GSEA enrichment results of eight hub genes. (**A**) NEDD8. (**B**) HIF1A. (**C**) SKP1. (**D**) BTRC. (**E**) CAND1. (**F**) CUL2. (**G**) UBE2S. (**H**) USP15

To explore the potential biological functions of NEDD8, HIF1A, SKP1, BTRC, CAND1, CLU2, UBE2S and USP15, we conducted single-gene GSEA analysis. These gene function enrichment results mainly include the allograft rejection, IL6/JAK/STAT3 signaling, inflammatory response, epithelial mesenchymal transition and TNF-α signaling via NFκB (Fig. 10).

### The expression of USP15 and CUL2 in COPD patients and cell lines

**Fig. 11:**
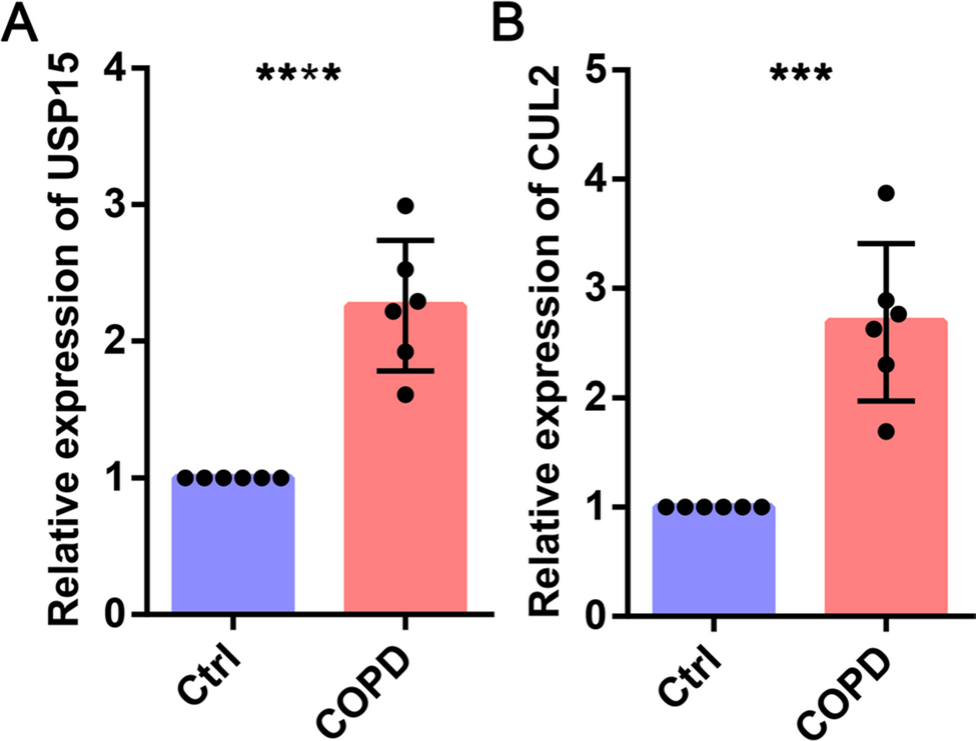
qPCR was used to detect the expression of USP15 and CUL2 genes in COPD patients and healthy samples. (**A**) USP15. (**B**) CUL2

**Fig. 12:**
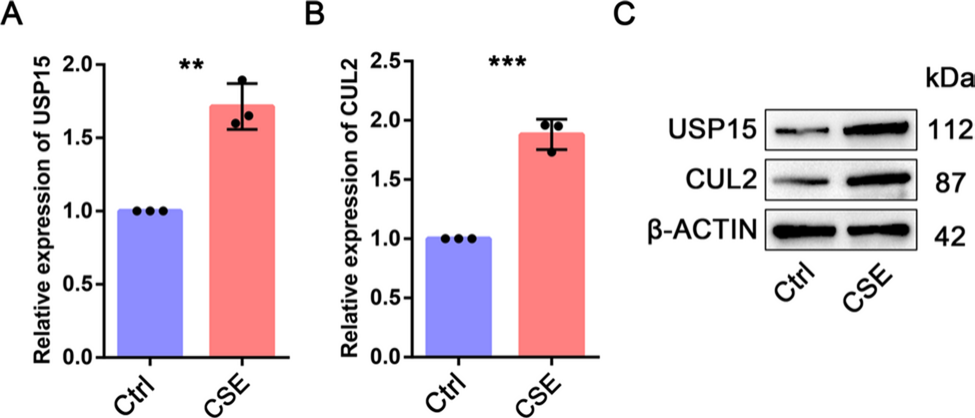
qPCR and western blot experiments were performed to detect the expression of USP15 and CUL2 in COPD cells and control cells. (**A**-**B**) The mRNA expression of USP15 and CUL2. (**C**) The protein expression of USP15 and CUL2

After searching various databases, we found that some hub genes had already been reported in COPD, while USP15 and CUL2 had never been explored in COPD. Therefore, we chose USP15 and CUL2 for subsequent experimental validation. We detected the mRNA expression of USP15 and CUL2 in COPD patients using qPCR. The results indicated that the expression of USP15 and CUL2 in COPD patients was higher than that in healthy control individuals (Fig. 11). We then examined the expression of USP15 and CUL2 in 16HBE cells exposed to CSE. The qPCR and western blot results indicated that the expression of USP15 and CUL2 in the CSE-treated group was higher than that in the control group (Fig. 12). Our expression validation results in COPD patients and cell models are consistent with the results of bioinformatics analysis.

## Discussion

The risk factors for COPD include genetic susceptibility, inflammation, and environmental factors [12–14]. In recent years, COPD has received increasing attention, treatments such as bronchodilators or anti-inflammatory drugs have been widely applied [15, 16]. However, the treatment of COPD needs improvement. Many studies have focused on the epigenetic aspects of COPD, such as RNA methylation or post-translational modifications of proteins. One study reported that ZC3H13 enhances the expression and mRNA stability of ITGA6 through m6A modification, affecting inflammation and fibrosis in bronchial epithelial cells in COPD [17]. Another study suggested that human epididymal protein 4 promotes the release of IL-6 in HBE cells by phosphorylating NFκB-p65, exacerbating airway inflammation and remodeling in COPD [18].

In this study, we identified 2,932 differentially expressed genes between COPD patients and healthy controls (Fig. 1). We further analyzed and identified 96 differentially expressed ubiquitination related genes, including 38 upregulated genes and 58 downregulated genes (Fig. 3 and 4). We subsequently performed GO and KEGG analyses on the 96 ubiquitination related genes. The results indicated that these genes were mainly enriched in post-translational protein modification, ubiquitin ligase complex and ubiquitin mediated proteolysis (Fig. 5 and 6). The ubiquitination process is one of the critical cellular functions and plays an important role in COPD. For example, the E3 ubiquitin ligase Pellino-1 can ubiquitinate the K63 site of p21, influencing lung cell senescence and the progression of COPD [19]. Moreover, LincR-PPP2R5C was found to regulate the ubiquitination of IL-1β in macrophages and promote airway inflammation and emphysema in a COPD mouse model [20]. Another study suggested that CSE enhances the degradation of STK11 protein in airway epithelial cells via the FBXL19-mediated ubiquitin-proteasomal pathway, leading to augmented cell death [21]. Overall, ubiquitination plays an important role in COPD. However, there are still many potential mechanisms that need to be explored.

Through PPI analysis, we found that the proteins encoded by these ubiquitination related genes interact with each other (Fig. 7). According to the results in Fig. 7B, the top 15 genes are defined as hub genes. These hub genes influence the pathological progression of different diseases through ubiquitination. In esophageal squamous cell carcinoma, Circ_0001821 affects cell proliferation and the cell cycle by enhancing BTRC-mediated IKBA ubiquitination [22]. Moreover, deubiquitinating enzyme USP25 improves myocardial ischemia-reperfusion injury by deubiquitinating NLRP3 and negatively regulating the activity of the NLRP3 inflammasome in cardiomyocytes [23]. In bladder cancer, the deubiquitinating enzyme PSMD7 promotes tumor development by stabilizing RAB1A expression [24]. However, research on COPD-related ubiquitination genes is still insufficient and requires further exploration.

We presented the expression of these 15 hub genes through box plot (Fig. 8). In addition, we analyzed the expression correlation among these 15 hub genes (Fig. 9A). The red and blue circles in Fig. 9A represent the positive and negative correlations between the expression of two genes, respectively. We also analyzed the potential upstream transcription factors of these hub genes. The results in Fig. 9B present the regulatory network between hub genes and potential transcription factors. These potential transcription factors may regulate the development of COPD by transcriptionally activating or inhibiting the expression of hub genes. These transcription factors play important roles in a variety of diseases. A study found that the transcription factor RUNX2 promotes drug resistance in triple-negative breast cancer through the TGF-β pathway by regulating breast cancer stem cells [25]. Additionally, USF1 transcriptionally activated USP14 to drive atherosclerosis by promoting EndMT through the NLRC5/Smad2/3 axis [26]. Many transcription factors have not been reported in COPD, and we plan to explore their roles in the development of COPD in future studies.

We performed single-gene GSEA analysis on 8 hub genes, and the results suggested that these functions are important processes in the pathological development of COPD, such as allograft rejection, IL6/JAK/STAT3 signaling, inflammatory response, epithelial mesenchymal transition and TNF-α signaling via NFκB (Fig. 10). Inflammatory response and epithelial mesenchymal transition are risk factors for COPD [27, 28]. Previous studies have demonstrated that miR-186-5p modulates the inflammatory response in COPD by targeting HIF-1α [29]. In addition, a recent study demonstrated that GLUT3-mediated cigarette smoke-induced epithelial mesenchymal transition in chronic obstructive pulmonary disease through the NF-kB/ZEB1 pathway [30]. It is necessary for us to study the impact of hub genes on the progression of COPD through inflammatory response or epithelial mesenchymal transition.

Based on bioinformatics analysis and literature search, we selected USP15 and CUL2 for subsequent experimental validation for the following reasons. First, some hub genes have already been explored in COPD, but USP15 and CUL2 have not yet been reported. Second, USP15 and CUL2 are ranked relatively high among the hub genes. The results of qPCR and western blot indicated that the mRNA and protein levels of USP15 and CUL2 were increased in the COPD group compared to the control group (Fig. 11 and 12). USP15 is a member of the ubiquitin specific protease (USP) family of deubiquitinating enzymes. USP15 mainly removes ubiquitin from target proteins and regulates various pathways, such as TGF-β receptor signaling and NF-kB signaling [31, 32]. CUL2 is a core component of the cullin-RING ubiquitin ligase complex, which is responsible for mediating the ubiquitination of target proteins [33, 34]. Multiple studies have reported that USP15 and CUL2 can influence the development of various diseases by modulating the ubiquitination of target proteins [35, 36]. Based on the experimental results and literature reports, we speculate that USP15 and CUL2 may influence the development of COPD through ubiquitination.

Multiple investigations have reported that ubiquitination is involved in various respiratory diseases. In lung adenocarcinoma, BZW2 promotes tumor malignant progression by enhancing the ubiquitination and degradation of GSK3β [37]. In asthma, the E3 ubiquitin ligase March1 promotes the expression of OX40L in allergen-stimulated dendritic cells by mediating the ubiquitination of HDAC11 [38]. Furthermore, RNF130 prevents pulmonary fibrosis by inhibiting aerobic glycolysis through mediating the ubiquitination of c-myc [39]. These published studies have demonstrated that ubiquitination plays an important role in various respiratory diseases.

Multiple articles have analyzed the potential molecular mechanisms of COPD from different perspectives using the CEO database, such as ferroptosis-related genes and pyroptosis related genes [40, 41]. Nevertheless, the association between ubiquitination related genes and COPD remains unreported. Our research has expanded our understanding of the development of COPD, however, there are still several limitations in this study. First, we validated the expression of genes and proteins at both clinical and cellular levels; however, the number of clinical samples included in this study was insufficient. We plan to collect more clinical samples to verify our conclusions. Moreover, we have identified several ubiquitination related genes associated with COPD, but the functions of these genes in COPD have not yet been explored. We plan to investigate the detailed mechanisms of USP15 and CUL2 in COPD.

In conclusion, our bioinformatics analysis identified 96 ubiquitination related genes in COPD, including 38 upregulated genes and 58 downregulated genes. In addition, our experiment results revealed that USP15 and CUL2 are upregulated in both clinical samples and cell lines of COPD, which is consistent with our bioinformatics findings. We believe that USP15 and CUL2 may play a role in the development of COPD through ubiquitination modification. Our results may provide new insights into the diagnosis and therapy of COPD.

## Electronic supplementary material

Below is the link to the electronic supplementary material.

Supplementary Material 1

Supplementary Material 2

Supplementary Material 3

## Data Availability

Publicly available datasets were analyzed in this study. The datasets GSE38974 for this study can be found here: https://www.ncbi.nlm.nih.gov/geo/.

## Abbreviations

BP: Biological process
CC: Cellular component
GEO: Gene expression omnibus dataset
GO: Gene Ontology
KEGG: Kyoto Encyclopedia of Genes and Genomes
MF: Molecular function
PPI: Protein-protein interactions
GSEA: Gene set enrichment analysis
TF: Transcription factor
