# Supplementary figures and images for: Identification and validation of USP15 and CUL2 as ubiquitination related biomarker in chronic obstructive pulmonary disease
Source: Hereditas. 2025 May 24;162:86. doi: 10.1186/s41065-025-00460-1 (PMC12103031; doi:10.1186/s41065-025-00460-1)

## Slide 1
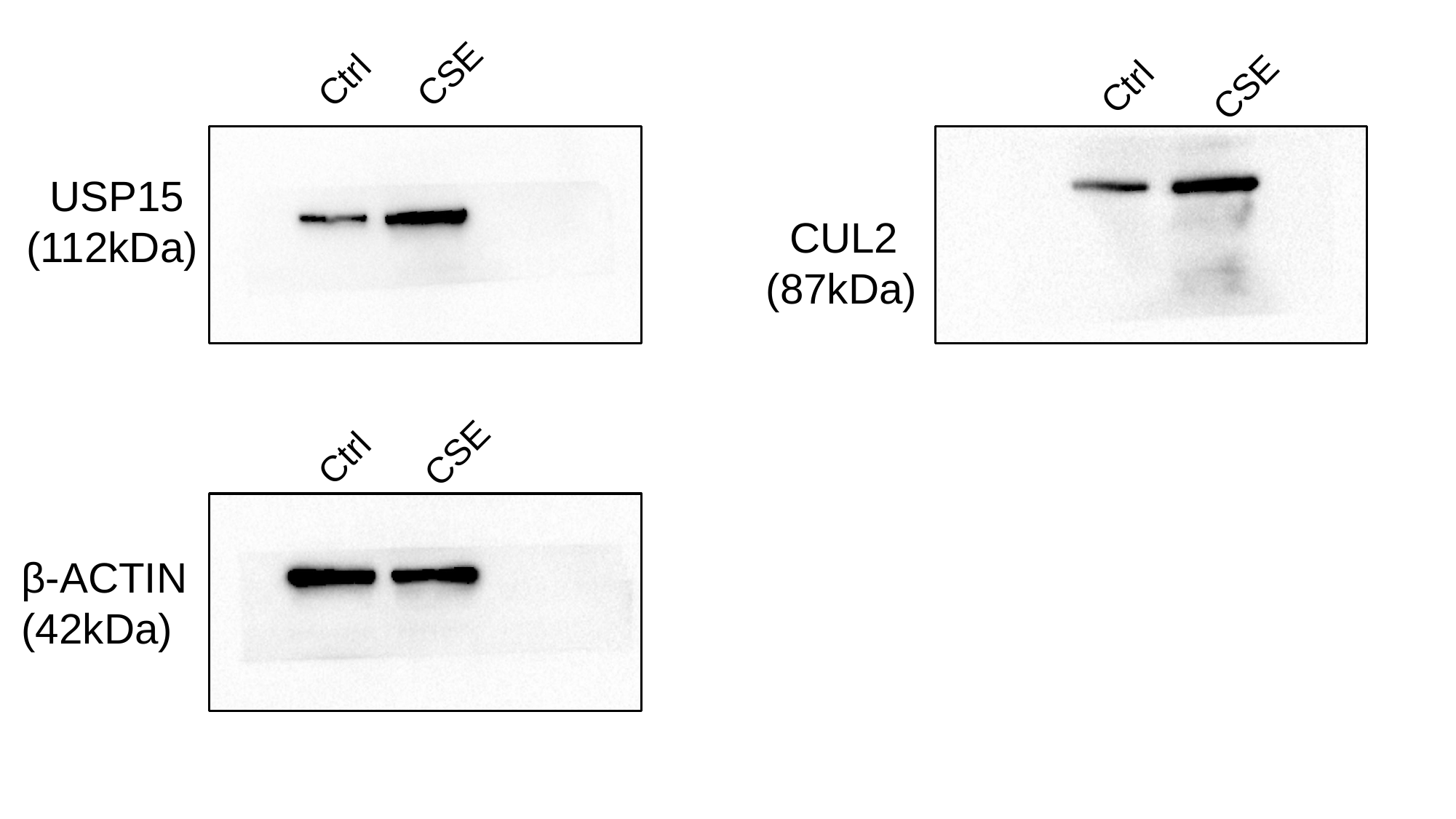

CSE
Ctrl
Ctrl
CSE
 USP15
(112kDa)
 CUL2
(87kDa)
CSE
Ctrl
β-ACTIN
(42kDa)

Supplement: Supplementary file 3 — Supplementary Material 3 [file 41065_2025_460_MOESM3_ESM.pptx]
